# Social Isolation Blunted the Response of Mesocortical Dopaminergic Neurons to Chronic Ethanol Voluntary Intake

**DOI:** 10.3389/fncel.2016.00155

**Published:** 2016-06-14

**Authors:** Valeria Lallai, Letizia Manca, Laura Dazzi

**Affiliations:** Department of Life and Environmental Sciences, Section of Neuroscience and Antropology, Centre of Excellence for the Neurobiology of Dependence, University of CagliariCagliari, Italy

**Keywords:** mPFC, social isolation, ethanol, dopamine, stress, psychological

## Abstract

Previous studies have shown that stress can increase the response of mesolimbic dopaminergic neurons to acute administration of drugs of abuse included ethanol. In this study, we investigated the possible involvement of the mesocortical dopaminergic pathway in the development of ethanol abuse under stress conditions. To this aim we trained both socially isolated (SI) and group housed (GH) rats to self administer ethanol which was made available only 2 ha day (from 11:00 to 13:00 h). Rats have been trained for 3 weeks starting at postnatal day 35. After training, rats were surgically implanted with microdialysis probes under deep anesthesia, and 24 hlater extracellular dopamine concentrations were monitored in medial prefrontal cortex (mPFC) for the 2 hpreceding ethanol administration (anticipatory phase), during ethanol exposure (consummatory phase) and for 2 hafter ethanol removal. Results show that, in GH animals, dopamine extracellular concentration in the mPFC increased as early as 80 min before ethanol presentation (+50% over basal values) and remained elevated for 80 min during ethanol exposure. In SI rats, on the contrary, dopamine extracellular concentration did not show any significant change at any time point. Ethanol consumption was significantly higher in SI than in GH rats. Moreover, mesocortical dopaminergic neurons in SI animals also showed a decreased sensitivity to an acute administration of ethanol with respect to GH rats. Our results show that prolonged exposure to stress, as in social isolation, is able to induce significant changes in the response of mesocortical dopaminergic neurons to ethanol exposure and suggest that these changes might play an important role in the compulsivity observed in ethanol addiction.

## Introduction

In the research field of drug addiction the mesolimbic dopaminergic system has always received great attention since the evidence that most of the substances of abuse are able to increase dopamine extracellular concentration in the Nucleus Accumbens (Di Chiara and Imperato, 1988). Only recently it has been suggested that also dopaminergic neurons projecting to the medial prefrontal cortex (mPFC) might play a crucial role in the development of dependence (for reviews, see Kalivas and Volkow, 2005; Koob and Volkow, 2010; Jentsch et al., 2014). mPFC is part of the mesocorticolimbic dopaminergic system whose neurons project from the VTA also to the Nucleus Accumbens and Amydgala (Björklund and Dunnet, 2007). These areas together form the reward and motivation circuitry which is crucial in the regulation of functions that are altered in drug addiction, such as attribution of incentive salience to a stimulus (Robinson and Berridge, 1993), activation of goal-directed behaviors (Salamone et al., 2007), evaluation of reward (Koob and Volkow, 2010). In this circuitry, the mesocortical dopaminergic pathway has been proved to be fundamental in regulating impulsivity and action inhibition (Jentsch et al., 2014), which are key feature in all the stages of drug addiction. Accordingly, disruption of the processes that regulate inhibitory control and reward sensitivity has been suggested to be important mechanism in the development of addiction which has been proposed to involve neuroadaptive changes in the mesocorticolimbic circuitry that in turn alter the mechanisms that regulate reward, motivation, memory consolidation, sensitivity to stress, executive and inhibitory control (Koob and Volkow, 2010).

The ventromedial PFC is highly involved in the evaluation of reward and in the process of decision making (Peters and Büchel, 2011). Thus, PFC dysfunction may exacerbate the loss of control associated with compulsive drug use and facilitate the progression to drug addiction (Jentsch et al., 2014; Koob et al., 2014). During abstinence from alcohol, mPFC functionally disconnects from the Amygdala while retaining connection to the Nucleus Accumbens; this functional disconnection has been suggested to be crucial for impaired executive control over motivated behavior suggesting that disregulation of mPFC interneurons may be an early index of neuroadaptation in alcohol dependence (Koob et al., 2014).

In the development of addiction, stress is known to be a key factor, which can increase the vulnerability to drug abuse (Koob et al., 2014). Accordingly, in a model of chronic exposure to stress, like social isolation at weaning, socially isolated (SI) rats show several evidences of a high propensity to addiction. Thus, SI rats have been shown to be more prone to self-administer amphetamine (Bardo et al., 2001; Whitaker et al., 2013), cocaine (Howes et al., 2000) and ethanol (Hall et al., 1998; Lodge and Lawrence, 2003; Deehan et al., 2007; McCool and Chappell, 2009; Whitaker et al., 2013; Lesscher et al., 2015).

In light of these evidences, in our study we evaluated the possibility that social isolation, as a model of chronic stress exposure, might induce a change in the sensitivity of mesocortical dopaminergic neurons to ethanol exposure which, in turn, would increase the vulnerability of SI rats to develop ethanol addiction. We hypothesize that a decreased response of mesocortical dopaminergic neurons to ethanol would induce a loss of control and a compulsive behavior toward drug use and facilitate the progression to drug addiction.

In our study, we evaluated the effect of both acute and chronic administration of ethanol to test whether the ethanol-induced response of mesocortical dopaminergic neurons might be different after acute or chronic administration of the drug. Moreover, to evaluate whether a decrease in prefrontal cortical function was associated with a vulnerability to ethanol addiction, we also measured the amount of ethanol consumed during self-administration protocol in SI and group housed (GH) rats, as a possible index of a higher propensity of SI animals to develop dependence from the drug.

## Materials and Methods

### Animals

Male Sprague Dawley CD young-adult rats (Charles River, Como, Italy) were bred in our animal facility and maintained under an artificial 12 h-light, 12 h-dark cycle (lights on from 08:00 to 20:00 h), at a constant temperature of 22 ± 2°C, and a relative humidity of 65%. They had free access to water and standard laboratory food at all times. All efforts were made to minimize animal suffering. Animal care and handling throughout the whole experimental procedures were made in accordance with the European Communities Council Directive of 24 November 1986 (86/609/EEC). The experimental protocols were also approved by the Animal Ethics Committee of the University of Cagliari and by the Italian Ministry of Health (authorization #353/2015-PR).

### Social Isolation and Voluntary Consumption of Ethanol Paradigm

At weaning, at postnatal day (PND) 21, the animals were housed individually SI or in groups of five per cage GH. From 28 PND to rats from both groups an ethanol solution was made available for self administration for 3 weeks, 2 h a day (from 11:00 to 13:00 h). To instigate ethanol reinforcement without food or fluid deprivation we used a modified initiation procedure (Samson et al., 1999) that involved the use of sucrose in the ethanol solution; sucrose concentration was progressively decreased, contextually keeping constant that of ethanol, according to the following paradigm: (days 1–2) 5% (v/v) ethanol + 5% (v/v) sucrose; (days 3–4) 5% ethanol + 4% sucrose; (days 5–6) 5% ethanol + 3% sucrose; (days 7–8) 5% ethanol + 2% sucrose; (day 9-end of treatment) 5% ethanol + 1% sucrose. By day 11 of treatment to the beginning of the experiment, the solution was kept constant to 5% ethanol (v/v) and 1% sucrose. Both SI and GH animals were placed in individual cages for the 2 hof daily exposure to ethanol to allow a precise measure of ethanol consumption. The weight of each rat, the amount of fluid (both water and ethanol), and food intake were monitored daily at the end of the session.

### Surgery and Experimental Procedures

Rats were anesthetized by intraperitoneal (i.p.) injection of chloral hydrate (0.4 g/kg), and a concentric dialysis probe was inserted at the level of the mPFC (A +3.2, ML +0.8, V −5.3 relative to the bregma), according to the Paxinos and Watson (1982) Atlas. The active length of the dialysis membrane (Hospal Dasco, Bologna, Italy) was restricted to 4 mm. As previously described (Dazzi et al., 2014), the length of the dialitycal membrane allowed to sample from both infralimbic and prelimbic cortices. Experiments were performed in freely moving rats, 24 hafter probe implantation to allow recovery from surgery procedures. Ringer’s solution [3 mM KCl, 125 mM NaCl, 1.3 mM CaCl_2_, 1 mM MgCl_2_, 23 mM NaHCO_3_, 1.5 mM potassium phosphate (pH 7.3)] was pumped through the dialysis probe at a constant rate of 2 μl/min. Samples of dialysate were collected every 20 min from 8:30 to 15:00 h and immediately analyzed for dopamine by high performance liquid chromatography (HPLC) with electrochemical detection as previously described (Dazzi et al., 2002b); the detection limit for dopamine was 2 fmol per injection. The average neurotransmitter concentration in the first two samples was taken as 100%, and all subsequent values were expressed as mean ± SEM relative to the basal value. The mean *in vitro* recovery of the probes was 15 ± 3%. All probes were tested before implantation, and those with a recovery value outside of this range were not used. The absolute concentration of dopamine was not corrected for this value. At the end of each experiment, the placement of the probe was verified histologically. All rats in which the probe was located outside of the target region were excluded from the analysis. A group of rats were subjected to an acute treatment with ethanol (0.5, 2 g/kg, i.p., 20% solution v/v); the drug was injected after three stable samples (variation in dopamine concentrations less than 20%). For the acute ethanol administration experiments the average neurotransmitter concentration in the first three samples was taken as 100%, and all subsequent values were expressed as mean ± SEM relative to the basal value.

### Statistical Analysis

Data are presented as Mean ± SEM of at least five animals per group. Microdialysis data were compared among groups with one- or two-way analysis of variance (ANOVA) for repeated measures, factors being treatment and time points. The raw values of dopamine concentration were used for the analysis, with absolute basal concentrations given in “Results” Section. Normal distribution of data was verified by Skewness and Kurtosis evaluation with Graph Pad Prism 5.0. *Post hoc* comparisons were performed with Newman-Keuls test. A *p* value of <0.05 was considered statistically significant for all experiments.

## Results

### Basal Extracellular Concentration of Dopamine in SI and GH Rats

**Figure 1:**
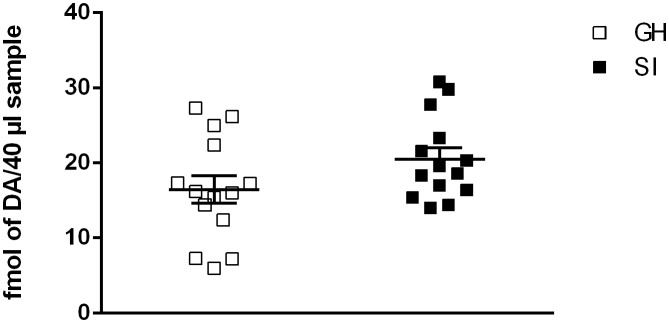
**Effect of social isolation on basal dopamine extracellular concentration.** Basal levels represent Mean ± SEM of 14 rats per group and are expressed as fmol of dopamine for 40 μl sample.

Basal extracellular concentration of dopamine in the mPFC of SI rats was not significantly different from that of GH rats (14.86 ± 2.926 fmol per 40 μl sample for GH rats vs. 20.22 ± 3.048 fmol per 40 μl sample for SI rats; Figure 1). One way ANOVA revealed a not significant effect between the two experimental groups [*F*_(1,28)_ = 0.60; *P* = 0.4498].

### Effect of Acute Administration of Ethanol on Extracellular Dopamine Concentration in the mPFC in SI and GH Rats

**Figure 2:**
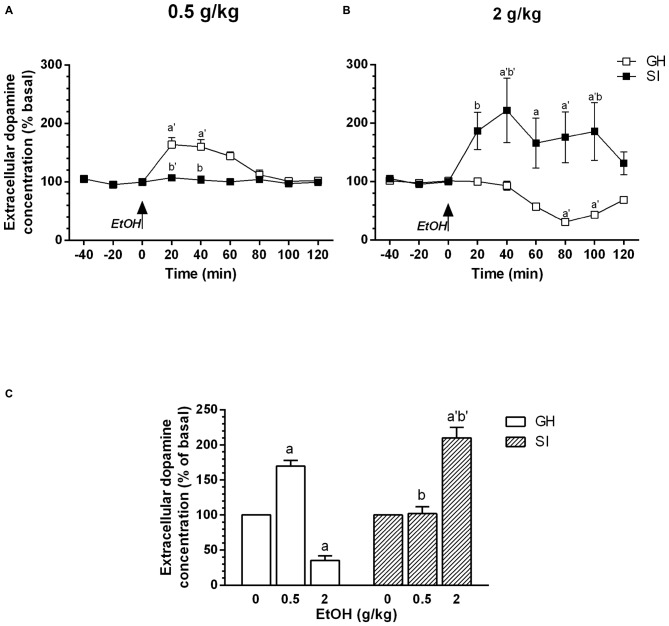
**Effect of acute administration of ethanol on extracellular dopamine concentration in the medial prefrontal cortex (mPFC).** Group housed (GH; □) or socially isolated (SI; ▪) animals received an acute administration of ethanol (0.5 or 2 g/kg, i.p., 20% v/v, **A,B**, respectively). **(C)** Shows the dose-response effect for GH and SI rats. Data are expressed as a percentage of basal values and are Mean ± SEM of at least 5 rats per group. ^a^*p* < 0.05; ^a’^*p* < 0.01 vs. basal value; ^b^*p* < 0.05; ^b’^*p* < 0.01 vs. corresponding point of GH rats.

We have previously shown (Dazzi et al., 2002b), that acute administration of ethanol is able to induce a biphasic effect on dopamine extracellular concentration in the mPFC, with lower doses inducing an increase and higher doses a decrease, respectively, in dopamine output. In this article, to evaluate whether in SI rats mesocortical dopaminergic neurons show a different sensitivity to the acute administration of ethanol, we used the same doses we used in our previous article (0.5–2 g/kg, i.p.). The present observations confirm our previous data and show that, in GH animals, the acute administration of a low dose of ethanol (0.5 g/kg, i.p., 20% v/v) induced an increase in dopamine extracellular concentration in the mPFC that was maximal (+60%) 40 min after administration and returned to basal values after 120 min (Figure 2A). An higher dose of ethanol (2 g/kg, i.p., 20% v/v), on the contrary, induced a significant decrease in the same parameter (Figure 2B) with the maximal effect (−50%) observed 80 min after ethanol administration and values returning to basal after 120 min.

In contrast, in SI animals the acute administration of the lower dose of ethanol (0.5 g/kg, i.p.) failed to significantly modify basal dopamine extracellular concentration (Figure 2A) while the higher dose (2 g/kg, i.p.) induced a significant increase (+90% vs. basal values) in dopamine output 20 min after administration, reaching its maximum after 40 min. The increase persisted for 100 min after the injection and returned to basal values in 120 min (Figure 2B). The effect induced in SI animals by administration of the higher dose was similar to that observed in GH rats after injection of the lower dose of ethanol (0.5 g/kg, i.p.; Dazzi et al., 2002b; present data), which, on the contrary, failed to induce any significant change in dopamine output in SI animals.

Thus, social isolation induced a shift in the dose-response relation on the effect of ethanol on dopamine output in the mPFC (Figure 2C).

For the dose of 0.5 g/kg, two-way ANOVA revealed a significant effect over time [*F*_(8,64)_ = 1.89; *P* < 0.01]; a significant effect of treatment [*F*_(1,64)_ = 7.28; *P* < 0.01]; and a significant effect of the interaction between factors [*F*_(8,64)_ = 5.75; *P* < 0.0001].

For the dose of 2 g/kg, two-way ANOVA revealed a significant effect over time [*F*_(8,64)_ = 2.92; *P* < 0.01]; a significant effect of treatment [*F*_(1,64)_ = 8.83; *P* < 0.05]; and a significant effect of the interaction between factors [*F*_(8,64)_ = 6.12; *P* < 0.0001].

### Voluntary Ethanol Consumption in GH and SI Rats

**Figure 3:**
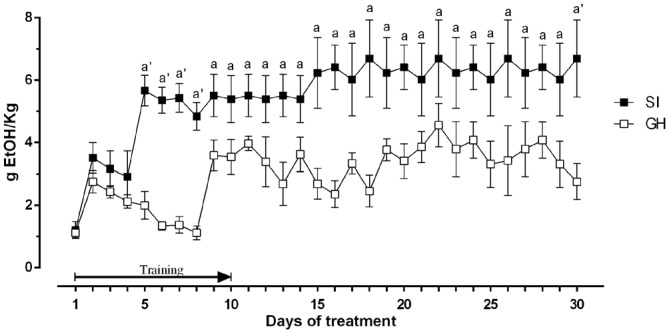
**Amount of ethanol consumed by rats trained in a voluntary intake protocol.** The ethanol containing solution was made accessible to the animals between 11:00 and 13:00, every day for 3 weeks. The first 10 days represent a training phase with constant concentration of ethanol and decreasing concentration of sucrose. The amount of ethanol consumed was calculated every day as difference between the weight of the bottles before and after consumption session and is expressed as Mean ± SEM of 20 rats per group. ^a^*p* < 0.05, ^a’^*p* < 0.01 vs. GH.

To evaluate whether a chronic stress exposure like social isolation might alter the preference for ethanol and/or the amount of the drug consumed, we measured the amount of ethanol consumed by SI and GH animals. As shown in Figure 3, there was a significant difference in voluntary ethanol consumption relative to housing history, starting from the 5th day of the training protocol with SI animals consuming a significantly greater amount of ethanol than GH rats. The amount of ethanol consumed reached a plateau at the 5th day of the treatment for SI rats (~6 g of ethanol/kg of body weight) and on the 9th day for GH (~3.8 g of ethanol/kg of body weight). Two-way ANOVA revealed a significant effect over time [*F*_(29,406)_ = 3.603; *P* < 0.0001]; a significant effect between the experimental groups [*F*_(1,20)_ = 12.55; *P* < 0.01]; and a not significant interaction between the factors [*F*_(29,406)_ = 1.130; *P* = 0.2962].

**Figure 4:**
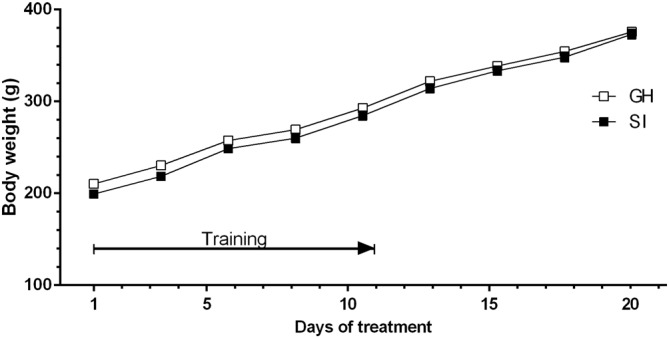
**Variations in the body weight of rats trained to a system of voluntary consumption of ethanol.** The solution was made accessible to the animals from 11:00 to 13:00, every day for 3 weeks. The body weights of both GH (□) and SI (▪) animals have been measured 3 days a week, for 3 weeks after removal of ethanol solution (at 13:00 h). Data are expressed as Mean ± SEM of 20 rats per group.

Figure 4 shows that body weight did not differ significantly between the two experimental groups during the voluntary ethanol intake protocol. SI and GH rats also showed a similar intake of total fluid and food (data not shown).

### Effect of Prolonged Voluntary Ethanol Intake on the Extracellular Concentration of Dopamine in the mPFC of SI and GH Rats

**Figure 5:**
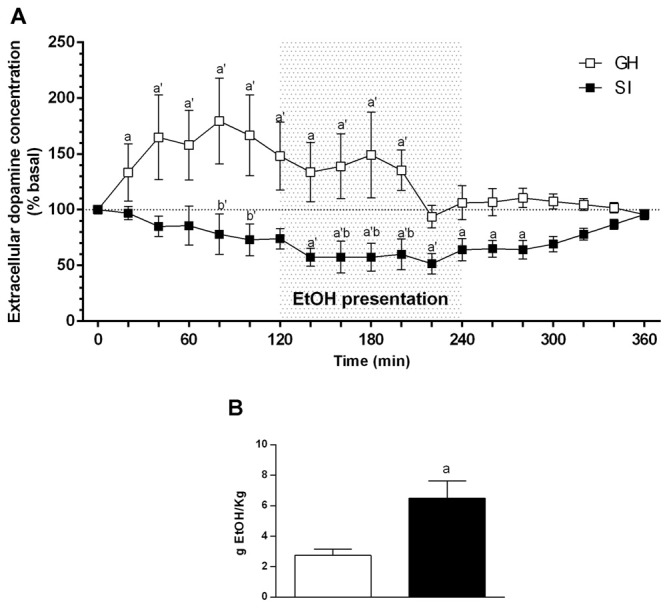
**Effect of social isolation on extracellular dopamine concentration in the mPFC during anticipation and consumption of ethanol after a voluntary ethanol intake protocol (A) and** **amount of ethanol consumed during the microdialysis experiment by SI and GH animals (B).** Animals have been trained to voluntary consume ethanol from 11:00 to 13:00 every day for 3 weeks. During this time the ethanol containing solution was made accessible to the animals, and microdialysis samples were collected from the mPFC before, during, and after ethanol presentation. The amount of ethanol consumed during the experiment was calculated at the end of the experiment as difference between the weight of the bottles before and after the consumption session and is expressed as Mean ± SEM of 20 rats per group. Data are expressed as a percentage of basal values and are Mean ± SEM of at least 5 rats per group. ^a^*p* < 0.05, ^a’^*p* < 0.01 vs. basal values; ^b^*p* < 0.05, ^b’^*p* < 0.01 vs. corresponding GH value.

In GH rats the chronic voluntary consumption of ethanol induced a significant increase of extracellular dopamine concentration as early as 120 min before ethanol presentation and increased further to reach a maximal value of +70% of basal values by 60 min before ethanol consumption. It then slightly declined to a value of +50% during ethanol intake to return to basal values 40 min before removal of the alcoholic solution (Figure 5A). In contrast, social isolation markedly reduced the sensitivity of mesocortical dopaminergic neurons to anticipation of ethanol. Indeed, in SI rats, in contrast to GH animals, the extracellular concentration of dopamine didn’t show any significant variation during the anticipatory phase; during the consummatory phase there was a slight but not significant decrease (−25% below basal values) in dopamine output. ANOVA revealed a significant effect over time [*F*_(18,198)_ = 1.628; *P* < 0.0001]; a significant effect of housing [*F*_(1198)_ = 9.130; *P* < 0.0001]; and a significant interaction between factors [*F*_(18,198)_ = 1.628; *P* < 0.05].

The amount of ethanol consumed by the two groups of rats during the experiment is shown in Figure 5B, and was significantly higher for SI than for GH rats (*P* < 0.01; Figure 5B).

## Discussion

Our results have shown that social isolation induces, in rats, a shift in the dose-response relation for the effect of the acute administration of ethanol in mesocortical dopaminergic neurons. In SI rats, in fact, the administration of 2 g/kg of ethanol, which in GH rats elicited a significant decrease in dopamine extracellular concentration in the PFC, induced an increase in the same parameter that was similar to the effect elicited by a lower dose (0.5 g/kg) in GH rats (present data; Dazzi et al., 2002b). The lower dose of ethanol failed to induce any significant effect in SI animals, while increased dopamine output in GH animals.

One possible explanation for the observed effect is that SI rats show a marked decrease in plasma and cerebrocortical concentrations of neuroactive steroids (Serra et al., 2000) and the effect of ethanol on mesocortical dopaminergic neurons is particularly sensitive to brain concentrations of progesterone and 3α, 5α-THDOC. In fact, an increase in the concentrations of these neurosteroids by prolonged administration of progesterone, is able to increase the response of mesocortical dopaminergic neurons to an acute administration of ethanol (Dazzi et al., 2002b). This effect was completely abolished by finasteride, which, by inhibiting 5α-reductase induces a marked decrease in plasma and brain concentration of 3α, 5α-THDOC (Concas et al., 1998; Dazzi et al., 2002a), suggesting that this progesterone metabolite is crucially involved in mediating this effect.

Alternatively, in SI rats the extracellular dopamine might be governed through a different mechanism or even circuitry with respect to GH animals.

The effect of ethanol on mesocortical dopaminergic neurons shown here is opposite to that recently observed by Karkhanis et al. (2014, 2015) who, instead, described an increase in dopamine response to acute ethanol in the Nucleus Accumbens and Amygdala of SI rats. Although discordant, these results are in agreement with the suggested alteration in the balance between the activity of mesocortical *vs* mesolimbic pathway in addiction (Kalivas and Volkow, 2005; Koob and Volkow, 2010), where an increased sensitivity to reward (Bonci et al., 2003), an increase in impulsivity and a decrease in inhibitory control (Jentsch et al., 2014) have been identified as key features to the development of addiction.

Accordingly, our data show that SI rats consumed significantly higher amounts of ethanol compared to their GH counterpart, suggesting that chronic exposure to stress, in a critical period like adolescence, might increase the vulnerability to develop addiction in this experimental group. The observation that SI and GH rats did not significantly differ in their body weight nor in the amount of food or total fluid consumed during the entire protocol, suggests that this effect is specific for ethanol.

Our results are in line with previous studies showing that social isolation increase voluntary ethanol intake (Hall et al., 1998; Lodge and Lawrence, 2003; Deehan et al., 2007; McCool and Chappell, 2009; Lesscher et al., 2015). However, this effect is strictly depending on the strain of rats used, on the drinking protocol and on the concentration of the ethanol solution used for the experiments, and some authors found a decrease in the amount of ethanol consumed by SI rats (Fahlke et al., 1997), and others found no effect (Ehlers et al., 2007).

We show that basal extracellular concentration of dopamine in the mPFC wasn’t significantly different in SI vs. GH rats; in fact, although there was a tendency toward an increase in this parameter, it did not reach statistical significance. These results are in agreement with previous microdialysis data (Dalley et al., 2002) but not with measurements of dopamine function in postmortem tissues of SI rats (Blanc et al., 1980; Jones et al., 1992; Heidbreder et al., 2000) that showed a significant increase in DOPAC/dopamine ratio in homogenates from the mPFC of SI vs. GH rats. However, as suggested by Dalley et al. (2002), microdialysis allows a more accurate measurement of the extracellular content of dopamine.

Our observation that in SI animals the increase in dopamine output induced by both anticipation and consumption of ethanol was dramatically blunted, together with the increased consumption of ethanol by rats in this experimental group, suggest that early and prolonged exposure to stress might increase the vulnerability to drug addiction. Previous evidence that social isolation didn’t change ethanol metabolism (Karkhanis et al., 2014), suggest that this effect may be due to a perturbation of the function of mesocortical dopaminergic neurons by chronic exposure to stress rather than to pharmacokinetic differences between the two experimental groups.

Our results showing that a blunted response of mesocortical dopaminergic neurons to ethanol is accompanied by an increase in the consumption of ethanol in SI rats, confirm the link between addictive behavior and dopaminergic hypofrontality. Together with the previous observation that in SI rats there is, on the contrary, an increase in the sensitivity of dopaminergic neurons in the nucleus accumbens and amygdala to ethanol (Karkhanis et al., 2014, 2015), they suggest that SI might be able to alter the balance between mesocortical and mesolimbic dopaminergic pathways, to increase vulnerability to addiction. A number of studies have in fact suggested that progression of addiction from a social use to compulsive use of a drug might be consequence of a decrease in the executive control and/or of a strengthening of the cortico-striatal circuitry that regulates habitual behavior. In fact, once a given behavior is acquired, the cortico-striatal-thalamic pathway allows the behavior to be efficiently performed without the activation of the prefrontal cortical circuitry (Jog et al., 1999; Canales, 2005) which is then able to integrate new information that can modify and drive the acquired behavior. Thus, by processing the environmental stimuli, the PFC has the ability to modify a given behavior whenever it results dangerous or inappropriate to the subject (Kalivas et al., 2008).

Addiction is characterized by the inability to stop or modify a behavior even when it clearly has negative consequences on the individual (Everitt and Robbins, 2005; Kalivas et al., 2008). Accordingly, a number of studies described a functional “hypofrontality” as a key feature in addiction (for review, see Jentsch and Taylor, 1999; Goldstein and Volkov, 2002; Jentsch et al., 2014). Together with the decline in frontal executive control, it has been shown a progressive strengthening of the compulsive behavior of seeking and taking the drugs (Everitt and Robbins, 2005). The latest researches on drug addiction have pointed out that drugs of abuse, as well as certain palatable foods, are able to induce neuroadaptive changes in the activity of the mesocorticolimbic circuitry (Volkow et al., 2003; Koob et al., 2014); in particular, in animals, withdrawal from drug addiction has been characterized by an increased responsiveness to reward and a decreased activity in the mesocortical dopamine system (Volkow et al., 2003). Our observation of a blunted sensitivity of mesocortical dopaminergic neurons to ethanol anticipation in SI rats suggests that chronic stress is able to reduce the response of this pathway to ethanol. This data, together with the previous observation that in SI rats mesolimbic dopaminergic neurons show, on the contrary, an increased response to acute ethanol administration (Karkhanis et al., 2014), further support the hypothesis that an important mechanism for the development of addiction is a disruption in the balance of the function between mesolimbic and mesocortical dopaminergic pathway. They also suggest that chronic exposure to social isolation stress, by blunting the sensitivity of mPFC projecting neurons to ethanol exposure, might increase the vulnerability to drug addiction. Our observation of a decreased efficacy of ethanol in SI rats already after a single administration, seems to suggest that the increased vulnerability is not dependent on previous ethanol exposure. Accordingly, in previous studies SI rats have shown greater increases in dopamine extracellular concentration in the Nucleus Accumbens after an acute administration of amphetamine (Hall et al., 1998), an enhanced Conditioned Place Preference for amphetamine and ethanol after a single conditioning session (Whitaker et al., 2013), an increased locomotor response to cocaine (Phillips et al., 1994), as well as an increased self-administration of drugs of abuse (Hall et al., 1998; Lodge and Lawrence, 2003; Deehan et al., 2007; McCool and Chappell, 2009).

In conclusion, our data show that chronic exposure to social isolation stress in a critical period such as early adolescence is able to modify the neurochemical response of mesocortical dopaminergic neurons to both acute and prolonged administration of ethanol. They suggest that a blunted sensitivity of mesocortical dopaminergic neurons might be a neuroadaptive adjustment to chronic stressful stimuli, which, in turn, would disrupt the balance between mesocortical and mesolimbic system’s function that has been suggested to be crucial for the development of addictive behavior (Kalivas and Volkow, 2005). Thus, ours and others result (Karkhanis et al., 2014), seem to suggest that both a decrease in mPFC responsiveness and an increased sensitivity in the Nucleus Accumbens and Amygdala to the effect of ethanol, are already present at the first administration of the drug, and that prolonged administration abolishes the motivational salience of ethanol anticipation, thus building in SI rats a vulnerability to ethanol addiction.

## Author Contributions

VL: performed the experiments, analyzed the data, wrote the article. LM: performed the experiments. LD: conceived and designed the experiments, analyzed the data, wrote the article.

## Conflict of Interest Statement

The authors declare that the research was conducted in the absence of any commercial or financial relationships that could be construed as a potential conflict of interest. The reviewer CSC and handling Editor declared their shared affiliation, and the handling Editor states that the process nevertheless met the standards of a fair and objective review.
